# Lower doses of isoflurane treatment has no beneficial effects in a rat model of intracerebral hemorrhage

**DOI:** 10.1186/1471-2202-14-129

**Published:** 2013-10-20

**Authors:** Elga Esposito, Emiri T Mandeville, Eng H Lo

**Affiliations:** 1 Neuroprotection Research Laboratory, Departments of Neurology and Radiology, Massachusetts General Hospital, Harvard Medical School, Boston, USA

**Keywords:** Anaesthesia, Neuroprotection, Brain edema, Intracerebral hemorrhage, Animal models

## Abstract

**Background:**

Intracerebral hemorrhage is a subtype of stroke that has a poor prognosis without an adequate therapy. Recently, the use of anesthetics such as isoflurane has been shown to be protective after cerebral ischemia. However, the potential therapeutic effect of isoflurane after intracerebral hemorrhage (ICH) has not been fully explored.

**Results:**

In this study, male Sprague–Dawley rats (SD) were subjected to ICH and randomized into controls and 1.2% or 1.5% isoflurane posttreatment groups. Brain water content, neurological outcomes and matrix metalloproteinase-2 and -9 (MMP2-MMP9) plasma levels were quantified at 24 hours. Isoflurane treatment did not reduce brain edema compared with controls in any of the applied isoflurane concentrations. Moreover, consistent with this lack of effect on brain edema, isoflurane posttreatment did not affect neurological outcomes in any of the tests used. Plasma MMP levels did not change.

**Conclusion:**

Our data suggested that there is no neuroprotection after isoflurane posttreatment in a rat model of ICH.

## Background

Intracerebral hemorrhage (ICH) is a subtype of stroke with the highest mortality of all stroke subtypes. ICH is characterized by extravasation of blood into the brain parenchyma with hematoma formation and concomitant edema and secondary brain damage [1,2]. The prognosis after ICH is poor and no widely effective treatments exist.

Recently, two endogenous neuroprotective mechanisms have been shown to be broadly effective after ischemic stroke: ischemic preconditioning and ischemic postconditioning [3,4]. These two strategies are thought to recruit natural adaptive responses that brain and other organs utilize to protect themselves from various insults. What is interesting is that many of the conditioning pathways are highly conserved, i.e. protection can be induced by a wide range of sublethal triggers [5,6]. Recently, isoflurane has been shown to be highly effective conditioning inducer in various animal models of cerebral injury. This may be translationally relevant since isoflurane can be easily applied in a clinical setting. In particular, isoflurane postconditioning had been widely demonstrated to be protective after the ischemia [7]. Here, we ask whether isoflurane may also have some neuroprotective effect when administrated after ICH.

## Methods

### Rat intracerebral hemorrhage model

All experiments were performed following protocols approved by Massachusetts General Hospital Institutional Animal Care and Use Committee in accordance with the National Institute of Health Guide for the Care and Use of Laboratory Animals. All procedures were performed in a blinded and randomized fashion. ICH was induced by collagenase injections. Male Sprague–Dawley rats (7 rats for each group) (Charles River Laboratories, Wilmington, MA) were anesthetized with isoflurane (1.2%) in 30%/70% oxygen/nitrous oxide. A catheter in the tail artery was used for measuring blood pressure, pH, PaO_2_, and PaCO_2_. Using a stereotactic frame, 1 μL of saline containing 0.5 U collagenase VII-S (Sigma) was injected through a 25-gauge needle over 5 minutes into the right striatum (from bregma: 1.2 mm anterior, 3.5 mm lateral, 5.3 mm depth). 10 minutes after the collagenase injection, rats were randomized into isoflurane (1.2% and 1.5% isoflurane through a face mask for 1 hour) versus control groups. All endpoints were assessed in a blinded manner.

### Measurement of brain edema

A standard wet–dry method was used for the measurement of total brain water content as an indicator of edema. At 24 hours after ICH, rats were euthanized and brains were removed immediately, without fixation. A single 4-mm section was cut centered around the hematoma, and edema was calculated as: [(wet weight - dry weight)/wet weight] × 100%.

### Gelatin zymography

Matrix metalloproteinase-2 and -9 levels in plasma were assessed with standard gelatin zymography. EDTA blood samples were immediately centrifuged at 10000 rpm for 10 minutes to obtain supernatants. Equal amounts of prepared protein samples were loaded and separated. After electrophoretic separation, the gel was renaturated and then incubated with developing buffer at 37°C for 20 hours. After developing, the gel was stained and then destained appropriately. Proteolytic bands in the zymography were quantified via densitometry.

### Neurological tests

The rats were assessed blindly at 24 hours using 3 tests: forelimb placement, body swing test, and a 5-point neuroscore scale. For the forelimb-placing test, animals were held close to a tabletop and scored for the ability to place the forelimb on the tabletop in response to whisker, visual, tactile, or proprioceptive stimulation. For the body swing test, animals were held approximately one inch from the base of their tails and elevated to an inch above a surface of a table. A swing was recorded whenever the rat moved its head out of the vertical axis to either side by more than 10° from vertical and then returned to the vertical position. Thirty total swings were counted per animal. Neuroscores were graded as 0 = no apparent deficit; 1 = slight deficit; 2 = circling; 3 = heavy circling or no movement at all; or 4 = death.

### Statistic analysis

Values are expressed as means ± S.D. Statistical analysis was performed with ANOVA, followed by Newman-Keuel’s test. Neurological outcomes (forelimb placement test, swing test, neuroscore) were analyzed using the nonparametric Kruskal-Wallis test. Statistical significance was accepted at the 95% confidence level (p < 0.05).

### ARRIVE (animal research: reporting in vivo experiments) guidelines

All experiments were performed following protocols approved by Massachusetts General Hospital Institutional Animal Care and Use Committee in accordance with the National Institute of Health Guide for the Care and Use of Laboratory Animals. All studies herein, including experimental design, animal care and use, randomization, blinding, statistical powering and analyses, inclusion/exclusion criteria, outcomes etc. are explicitly defined following ARRIVE guidelines.

## Results

**Figure 1:**
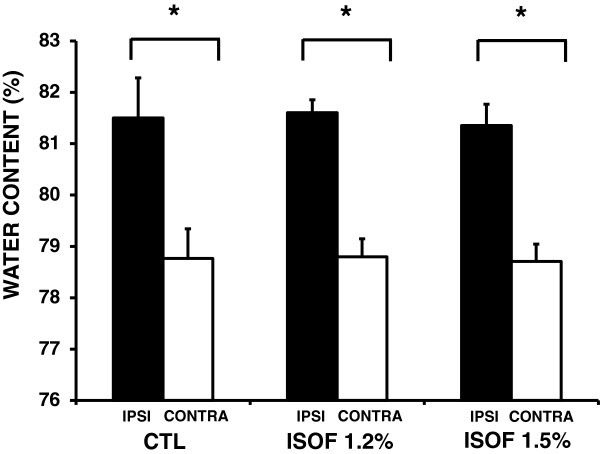
**Quantization of hemorrhagic edema after 24 hours.** Brain water content in the ipsilateral hemisphere was significantly greater than the contralateral hemisphere in both control and isoflurane groups *P < 0.05. However, ipsilateral water content was not affected by 1.2% and1.5% isoflurane. All values depicted as mean ± S.D.

As expected, this standard model of collagenase injections resulted in reproducible intrastriatal hematomas. There were no statistically significant differences in hematoma volumes in controls vs 1.2% or 1.5% isoflurane treatment groups. Accordingly, brain edema developed in all groups - brain water content in the ipsilateral hemisphere was significantly greater than the contralateral hemisphere (control group: ipsilateral, 81.5% ± 0.8% versus contralateral, 78.9% ± 0.4% *P* < 0.05; 1.2% isoflurane group: ipsilateral, 81.6% ± 0.2% versus contralateral, 78.8% ± 0.6% *P* < 0.05; 1.5% isoflurane group: ipsilateral, 81.3% ± 0.4% versus contralateral, 78.7% ± 0.3% *P* < 0.05). However, isoflurane treatment did not reduce brain edema compared with controls in any of the applied isoflurane concentrations (P = 0.653) (Figure 1).

**Figure 2:**
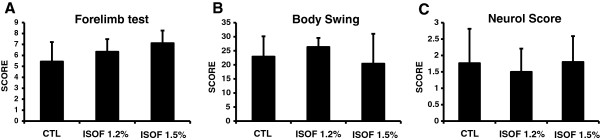
**Neurological outcomes were quantified at 24 hours in 1.2% and 1.5% isoflurane group compared with untreated controls.** There were statistically significant differences between any of the treatment groups for **(A)** the forelimb placement test, **(B)** body swing test, and **(C)** neuroscore. All values depicted as mean ± S.D.

Consistent with this lack of effect on brain edema, isoflurane posttreatment did not affect neurological outcomes in any of the tests used (Figure 2A,B,C). In the forelimb placement test, 1.2% and 1.5% isoflurane had no effect on the outcomes (control ipsilateral group, 5.43 ± 1.8; 1.2% isoflurane ipsilateral group, 6.33 ± 1.15; 1.5% isoflurane ipsilateral group, 7.1 ± 1.15. P = 0.096). Both 1.2% and 1.5% isoflurane posttreatment also had no detectable effect on the body-swing test (control group, 22.9 ± 7.2; 1.2% isoflurane group, 26.33 ± 3.2; 1.5% isoflurane group, 20.4 ± 10.6. P = 0.368). Finally, isoflurane did not affect neuroscore outcomes (control ipsilateral group, 1.76 ± 1.0; 1.2% isoflurane ipsilateral group, 1.5 ± 0.7; 1.5% isoflurane ipsilateral group, 1.8 ± 0.8. P = 0.773) or rates of mortality.

Isoflurane can lower blood pressure. In this study, blood pressure in the 1.5% isoflurane group was significantly lower than the 1.2% isoflurane and control groups. All other physiological parameters were within the normal range in all groups from the beginning of surgery until 1 hour after ICH induction (corresponding to the end of isoflurane treatments) (Table 1).

**Table 1:** Physiological parameters

	**Controls**	**Isof 1.2%**	**Isof 1.5%**
**MABP (mmHg)**			
before ICH	105.80±5.9	104.4±6.9	99.20±5.3
30′ after ICH	106.8±1.9	107.4±5.4	96.8±8.17
1 h after ICH	106.06±3.9	100.8±5.5	89.4±6.07
**pH**			
before ICH	7.47±0.001	7.44±0.017	7.44±0.034
30′ after ICH	7.41±0.020	7.42±0.046	7.42±0.017
1 h after ICH	7.41±0.017	7.41±0.021	7.39±0.036
**pO2**			
before ICH	159.80±7.26	190.60±46.71	167.8±17.4
30′ after ICH	158.4±23.04	180.2±21.79	155.6±36.88
1 h after ICH	159.60±15.34	189.80±8.44	159.00±7.84
**pCO2**			
before ICH	34.1±2.42	39.8±3.90	33.5±2.69
30′ after ICH	41.98±1.56	41.9±4.62	37.0±3.48
1 h after ICH	41.5±2.11	43.2±3.80	38.16±6.07

**Figure 3:**
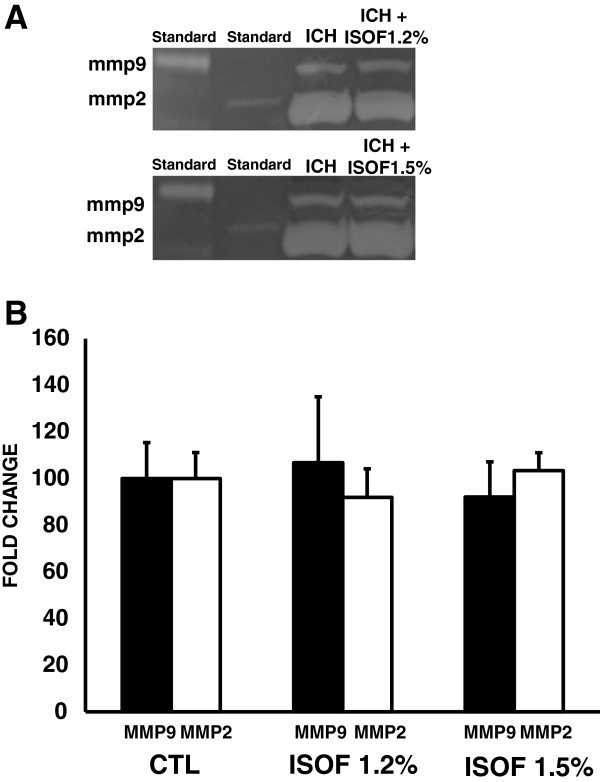
**Zymographic detection of MMP-2 and MMP-9 in plasma control and isoflurane groups. (A)** Representative gelatin zymography. **(B)** Densitometric quantization (mean ± S.D.) detected no differences in MMP-2 and MMP-9 plasma levels between the 1.2% and 1.5% isofurane groups vs the control group.

Gel zymography from plasma of rats post-treated with 1.2 or 1.5% isoflurane did not reveal any changes in matrix metalloproteinases MMP-2 or MMP-9 levels compared with control rats at 24 hrs after ICH (MMP9: control group, 100% ± 15.5; 1.2% isoflurane group, 106.86 ± 28.2; 1.5% isoflurane group, 92.14 ± 15.1. P = 0.539. MMP2: control group, 100% ± 15.1; 1.2% isoflurane group, 91.94 ± 12.3; 1.5% isoflurane group, 103.40 ± 7.6. P = 0.223) (Figure 3A,B).

## Discussion

In the last few years, many advances have been obtained in term of dissecting the signaling pathways activated by endogenous neuroprotective mechanisms after an ischemic insult [8]. Interestingly, it has been recently reported that ischemic preconditioning, a sublethal ischemic episode applied before a longer harmful ischemia [9], and ischemic postconditioning, a sublethal ischemia subsequent to a prolonged harmful ischemic episode, are both able to produce remarkable neuroprotection in various models of stroke and neuronal injury. However, preconditioning or postconditioning with an ischemic stimulus, although useful for proof-of-concept and dissecting molecular mechanisms, may be difficult to apply in a clinical setting. More recently, it has been suggested that conditioning may also be stimulated with various pharmacologic approaches. Emerging studies now suggest that the widely used anesthetic isoflurane can induce postconditioning and protect heart and brain against ischemia [10]. However, the potential therapeutic effect of isoflurane has not been fully explored after intracerebral hemorrhage (ICH). Here, we ask whether isoflurane, as showed during ischemic postconditioning, may also have some neuroprotective effect when administrated after ICH.

Two concentrations of isoflurane were tested in a well-established rat model of collagenase-induced ICH. Our data demonstrated that isoflurane posttreatment did not affect edema. Since neurovascular damage after ICH is closely related to MMP activation [11,12], we also assessed potential MMP biomarker levels in plasma. Once again, no effects of isoflurane were detected. Finally, consistent with the lack of morphological or biochemical effects, there were no differences in a wide battery of neurological tests at 24 hrs post-ICH. The observed lack of beneficial effects following isoflurane treatment in the present study may be due to the lower doses tested here. Blood pressure effects prevented us from assessing higher isoflurane concentrations. However, we cannot unequivocally exclude the possibility that higher doses may have had beneficial effects in this ICH model system.

In contrast to our negative results, Khatibi et al. [13] recently suggested that 1.5% isoflurane posttreatment significantly reduced peri-hematoma edema, ameliorated apoptotic cell death and rescued neurologic function in a different model of ICH. These different results could be related to a species difference (mice vs rats) or to the different methods used to produce ICH. In the previous Kathibi et al. study, ICH was induced by infusing autologous blood into the striatum of mice. In the present study, collagenase injections were used to produce cerebrovascular rupture and hemorrhage in rat brains. There are no perfect animal models of ICH. But the potential advantage of the collagenase model is that it replicates the primary vascular trauma and rupture that is present in human ICH [14]. However, a potential drawback is that compared to the blood infusion model, the collagenase approach may be associated with altered inflammatory responses [15].

## Conclusions

In conclusion, our study was unable to show a protective effect of isoflurane posttreatment in a rat model of collagenase-induced ICH. This negative study may be important because it suggests that, compared to cerebral ischemia, the beneficial actions of isoflurane posttreatment may not be as robust for other subtypes of stroke. Further studies are required to carefully test isoflurane in a wider spectrum of ICH animal models before any translation into clinical applications can be contemplated.

## Abbreviations

EDTA: Ethylenediaminetetraacetic acid; ICH: Intracerebral hemorrhage; MMP: Matrix metalloproteinase; SD: Sprague–dawley rats.

## Competing interests

The authors declare that they have no competing interest.

## Authors’ contributions

EE participated in the design of the study, drafted the manuscript and performed the experiments. ETM performed the randomization and provided assistance with data processing and analyses. EHL supervised the experimental design and the coordination of all the experiments, and edited the manuscript. All authors read and approved the final draft.
